# sRNAdb: A small non-coding RNA database for gram-positive bacteria

**DOI:** 10.1186/1471-2164-13-384

**Published:** 2012-08-10

**Authors:** Jordan Pischimarov, Carsten Kuenne, André Billion, Jüergen Hemberger, Franz Cemič, Trinad Chakraborty, Torsten Hain

**Affiliations:** 1 Institute of Medical Microbiology, Justus-Liebig-University, Schubertstrasse 81, Giessen, D-35392, Germany; 2 Institute for Biochemical Engineering and Analytics, University of Applied Sciences Giessen-Friedberg, Wiesenstrasse 14, Giessen, D-35390, Germany

## Abstract

**Background:**

The class of small non-coding RNA molecules (sRNA) regulates gene expression by different mechanisms and enables bacteria to mount a physiological response due to adaptation to the environment or infection. Over the last decades the number of sRNAs has been increasing rapidly. Several databases like Rfam or fRNAdb were extended to include sRNAs as a class of its own. Furthermore new specialized databases like sRNAMap (gram-negative bacteria only) and sRNATarBase (target prediction) were established. To the best of the authors’ knowledge no database focusing on sRNAs from gram-positive bacteria is publicly available so far.

**Description:**

In order to understand sRNA’s functional and phylogenetic relationships we have developed sRNAdb and provide tools for data analysis and visualization. The data compiled in our database is assembled from experiments as well as from bioinformatics analyses. The software enables comparison and visualization of gene loci surrounding the sRNAs of interest. To accomplish this, we use a client–server based approach. Offline versions of the database including analyses and visualization tools can easily be installed locally on the user’s computer. This feature facilitates customized local addition of unpublished sRNA candidates and related information such as promoters or terminators using tab-delimited files.

**Conclusion:**

sRNAdb allows a user-friendly and comprehensive comparative analysis of sRNAs from available sequenced gram-positive prokaryotic replicons. Offline versions including analysis and visualization tools facilitate complex user specific bioinformatics analyses.

## Background

In recent years numerous small non-coding RNAs (sRNAs) were discovered in bacteria. This class of RNAs is crucial to prokaryotic life, modulating transcription or translation leading to either activation or repression of important physiological processes. sRNAs enable bacteria to trigger rapid physiological responses in order to adapt to the environment or infectious processes [1-3].

To cope with the increasing number of identified sRNAs, databases such as fRNAdb, Rfam, sRNAMap and sRNATarBase were developed [4-9]. All of these approaches have certain drawbacks. fRNAdb contains all classes of RNAs, but allows no further analysis. Rfam is one of the most informative data collections, allowing detailed analyses via a web front-end. sRNAMap is a webserver-based application for gram-negative bacteria only. sRNATarBase compiles experimental data and allows the prediction of sRNA targets. But all databases available to date limit the analysis to published data only. Therefore bioinformatics analyses of candidate sRNAs in combination with genomes, terminators and other relevant information that has not yet been published is still a very complicated task.

In an attempt to overcome some of the aforementioned drawbacks, we have developed sRNAdb. Our database is a locally installable web-suite, permitting the comparative analysis of sRNAs of gram-positive bacteria including their flanking genes. User modified files in GenBank format and gram-negative bacterial genomes, pooled sRNA candidates or further features of interest can be included in locally installed databases. Furthermore all integrated analysis tools can also be used locally.

## Construction and content

**Figure 1:**
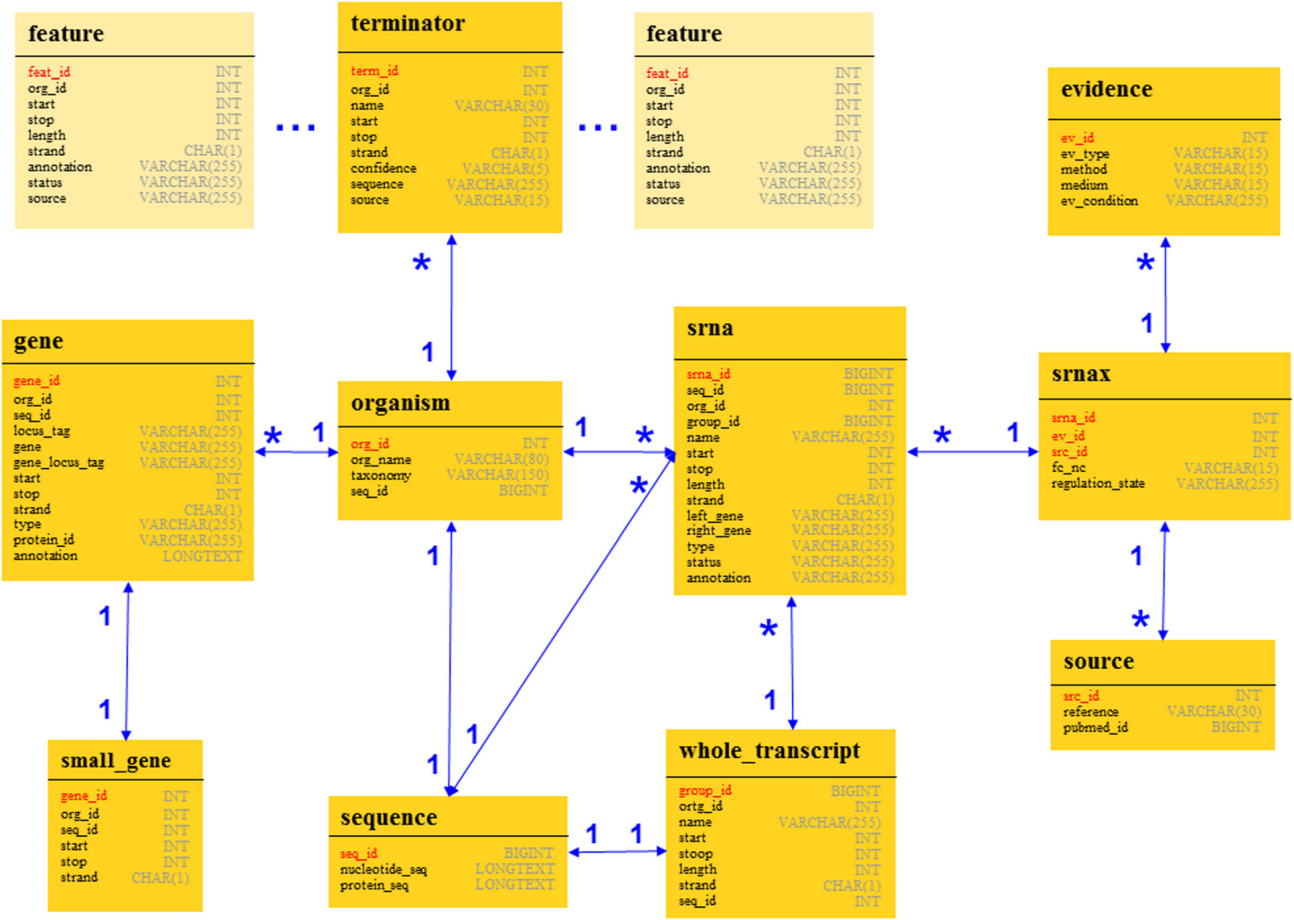
** Database schema.** The whole database with connections between tables and specific attributes are shown in UML-Notation. Unique and foreign keys of each table are given in bold letters while relations between entities are stated above the connection arrows. Optional features which can be inserted by the user into local versions of the database, are indicated using a lighter background color than employed for boxes representing entities.

A database scheme of unique keys and entities, combined with corresponding relations and connections is given in Figure 1. Optional user defined extensions to locally installed versions of the database are indicated with a lighter background color than the boxes representing database entities.

### Input data

To the best authors’ knowledge, no general nomenclature convention for sRNAs exists to date. Therefore sRNAs imported into our database from the literature cannot always be unambiguously distinguished by name, locus or annotation only. Furthermore a large number of published sRNAs is currently annotated as predicted or putative. This leads to a myriad of sRNAs bearing indistinct names, positions or ambiguous annotations. To cope with this difficulty, sRNAdb contains a unique key composed of information about the authors, experimental conditions and sRNA properties as shown in the table termed snrax of Figure 1. Annotated sequences of organisms or plasmids downloaded from NCBI’s RefSeq database [10] represent the replicons in the database. Information annotated in GenBank-formatted files such as sequences, or genes filtered from these files are automatically inserted into sRNAdb. When sRNAdb is installed locally, users can furthermore modify the local database by adding customized features such as terminators, promoters and other additional data. Terminators predicted by TransTermHP [11] serve as examples for this option, as described on the official sRNAdb server homepage.

### Architecture and design

Our public sRNAdb server is implemented in Java 1.6 on a Debian Linux PC. It facilitates a client–server architecture using Java Server Pages (JSPs), Java Servlets, and Cascading Stylesheets (CSS). Apache Tomcat and MySQL serve as webserver and database, respectively.

Related sRNAs are determined using BLASTN [12], while protein homologies are established by a combination of BLASTCLUST and BLASTP [12]. The addition of new data (replicons, sRNAs, terminators, promoters, RBS, etc.) to a local installation of sRNAdb is a simple process based on GenBank and tab-delimited flat-files.

Currently, the public sRNAdb server contains 558 gram-positive genomes and plasmids as well as 9993 automatically predicted and 671 experimentally verified sRNAs. An overview is given in Table 1.

**Table 1:** The table shows an overview of the current database entries. These are compiled from experiments or from bioinformatic analyses

**Reference**	**sRNAs**	**Organism**	**Pubmed_id**
Arnvig et al. 2009 [13]	9	*Mycobacterium tuberculosis* H37Rv	19555452
Bohn et al. 2010 [14]	28	*Staphylococcus aureus subsp. aureus* N315	20511587
Christiansen et al. 2006 [15]	3	*Listeria monocytogenes* EGD-e	16682563
D’Hérouel et al. 2011 [16]	22	*Enterococcus faecalis* V583	21266481
Geissmann et al. 2009 [17]	11	*Staphylococcus aureus* subsp. *aureus* N315	19786493
Irnov et al. 2010 [18]	90	*Bacillus subtilis* subsp. *subtilis* str. 168	20525796
Kumar et al. 2010 [19]	50	*Streptococcus pneumonia* TIGR4	20525227
Livny et al. 2008 [20]	9993	Gram-positive bacteria	18787707
Mandin et al. 2007 [21]	12	*Listeria monocytogenes* EGD-e	17259222
Mraheil et al. 2011 [22]	150	*Listeria monocytogenes* EGD-e	21278422
Nielsen et al. 2008 [23]	1	*Listeria monocytogenes* EGD-e	18621897
Perez et al. 2009 [24]	33	*Streptococcus pyogenes* MGAS5005	19888332
Rasmussen et al. 2009 [25]	84	*Bacillus subtilis* subsp. *subtilis* str. 168	19682248
Tezuka et al. 2009 [26]	12	*Streptomyces griseus* subsp. *griseus* NBRC 13350	19465662
Toledo-Arana et al. 2009 [27]	103	*Listeria monocytogenes* EGD-e	19448609
Vockenhuber et al. 2010 [28]	63	*Streptomyces coelicolor*	21521948

The organisms for which sRNAs are listed in the database, including references, the number of identified sRNAs for the specific organisms and their relevant pumed identification number are listed.

## Utility and discussion

**Figure 2:**
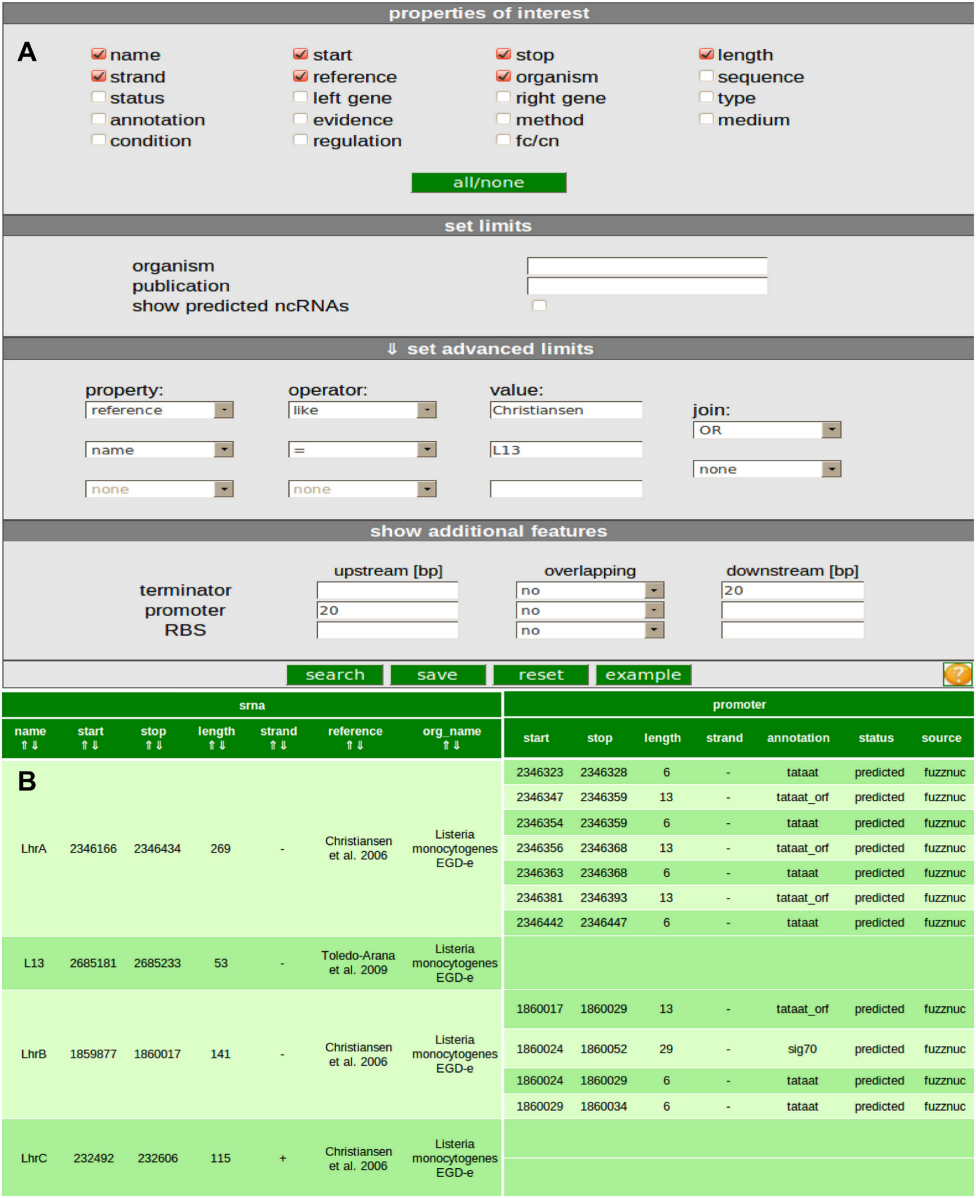
** Search servlet.** Properties of interest for each sRNA such as name, start, stop and so forth can be selected by setting check marks in the *properties* section of the servlet form. sRNAs of specific organisms or publications can be selected according to settings defined in the *set limits* section. Furthermore advanced limits for detailed filtering are available. Additional features like promoters and terminators can be searched for in the neighborhood of sRNAs of interest. **B** An example output from the *search* servlet. The resulting table contains four sRNAs named LhrA, LhrB, LhrC and L13. The corresponding search options are shown in **A**. For each sRNA, properties as well as additional features (promoters) in the surrounding area are displayed in intervals of 20 bp. Also the properties as selected with the *search* servlet are included in the output.

The sRNAdb web-database aims to collect all published and predicted sRNAs of gram-positive bacteria for comparative analysis. sRNAs featuring an environmental condition-depending range of sizes can optionally be joined to a combined transcript. The public version of sRNAdb contains terminators predicted by TranstermHP [11]. Three web-interfaces are provided for retrieval and analysis of the data. The first module is called *search* and offers a rich query interface for the database, as shown in Figure 2A. Properties of sRNAs can be selected and filters can be defined to create task-specific queries resulting in a tabular output (Figure 2B). Related or customized data can also be collated to the query, based on the up- or downstream distance to an sRNA of interest. Furthermore, a secondary structure prediction of selected sRNA sequences by energy minimization can be performed using RNAfold (http://rna.tbi.univie.ac.at/cgi-bin/RNAfold.cgi) .

**Figure 3:**
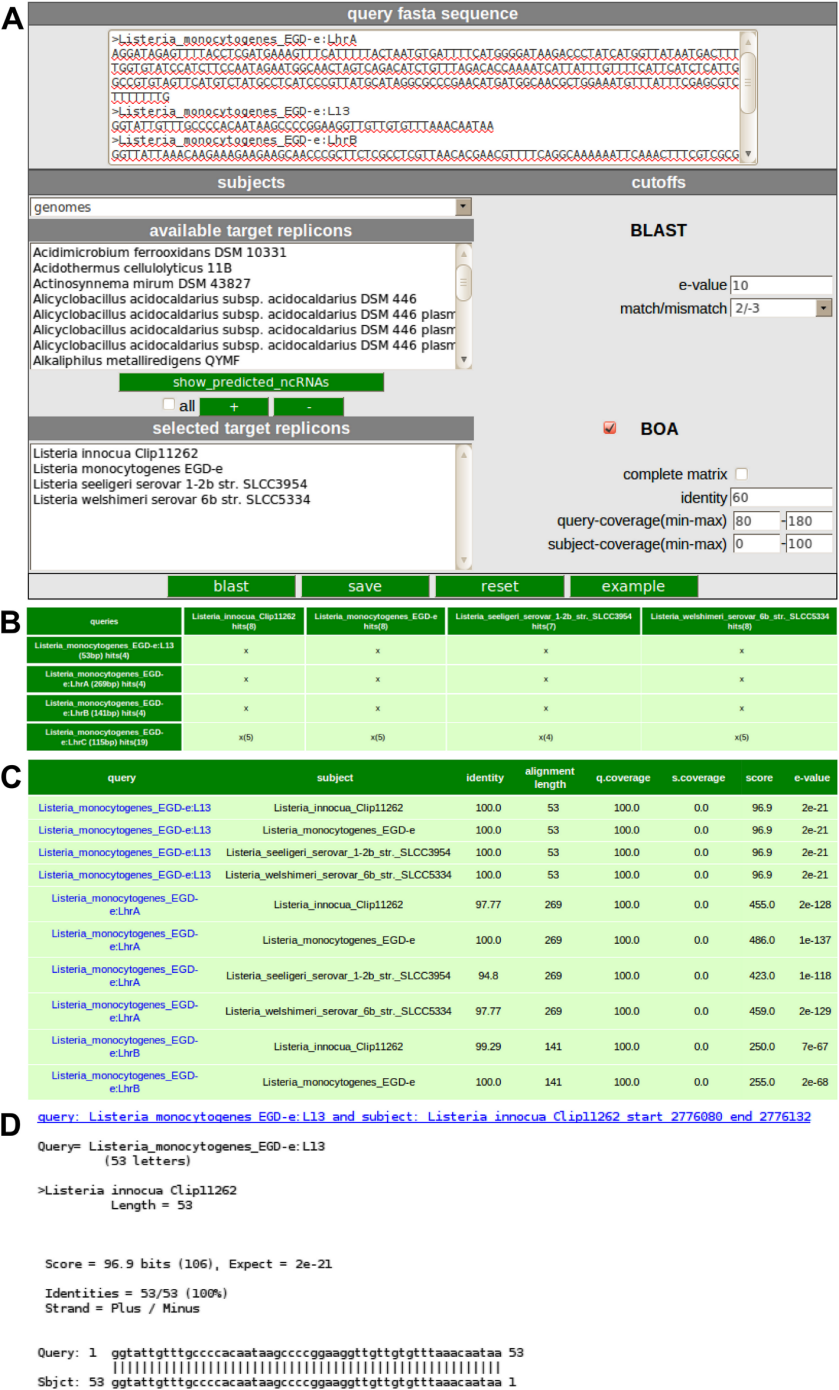
** Blast servlet form and corresponding output. A** FASTA formatted sRNA sequences can be inserted into the query box. Also target genomes or sRNAs have to be selected for multiple alignment using BLAST. For a detailed BLAST analysis the BLAST output analysis (BOA) options has to be selected. In this example four sRNAs resulting from a search with parameters shown in Figure 1 were selected as input. Genomes of the genus *Listeria* were set as targets and the BOA options were enabled. **B** The number of sRNAs detected in the target organism is displayed in a comparative matrix form. **C** All hits listed in a table and are linked to their corresponding alignment. **D** A detailed BLAST alignment of all results can also be plotted.

Another interface named *blast* (Figure 3A) was created to enable homology searches of sRNAs versus either public or proprietary sRNAs or whole chromosomes/plasmids using BLASTN [12]. This can be used for initial screening of potential genomic regions. Concise matrix outputs for comparative analysis purposes as shown in Figure 3B and Figure 3C, are implemented. Complete BLAST alignments are displayed in Figure 3D. Sequences from the BLAST output table can be easily selected by setting checkmarks to extract data into a multifasta-formatted file, ready to serve as input to multiple sequence alignment programs such as CLUSTALW (http://www.ebi.ac.uk/Tools/msa/clustalw2/). The resulting output can be used to predict structurally conserved and thermodynamically stable RNA secondary structures using e.g., RNAz (http://rna.tbi.univie.ac.at/cgi-bin/RNAz.cgi), facilitating screens for sRNA-homologs across genomes.

**Figure 4:**
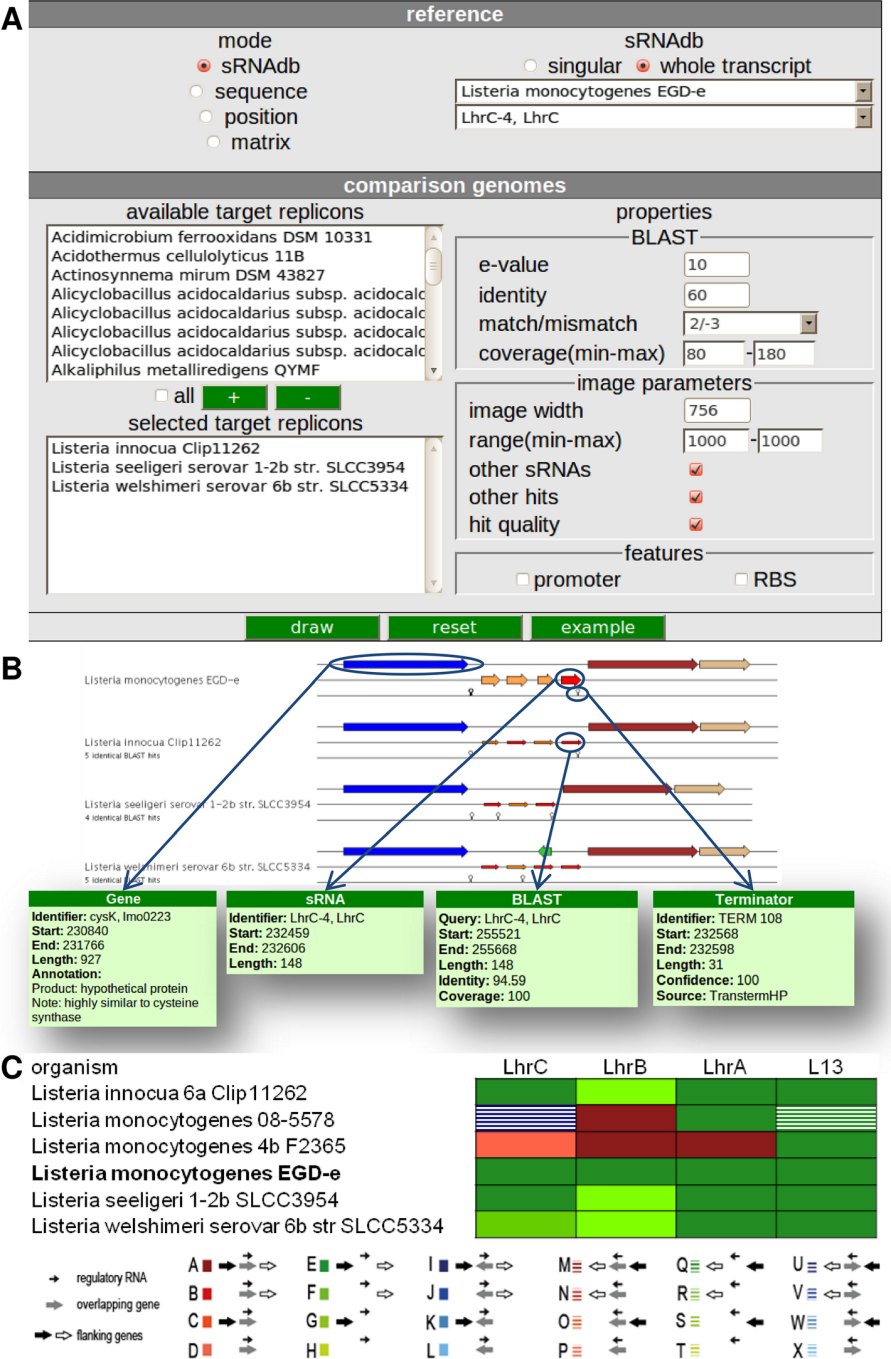
** Vision servlet forms and result of single and batch mode.** Different input options are available. After selecting the sRNA of interest, replicons can be selected for visualization. Options for further analyses based on BLAST, as well as properties relating to the image output can be set. **A** An example relating to the LhrC transcript is displayed. **B** Single mode: the resulting image shows a comparative representation of a single sRNA candidate and flanking genes in selected organisms. Moving the mouse pointer over these, the corresponding properties of each object is shown in a separate popup window. **C** Batch mode: sRNAs displayed in Figure 1 are used as input in this example. The output-matrix indicates occurrence of the sRNA candidates in selected organisms and their directional relationships with respect to their surrounding genes.

For comprehensive visual assessment the *vision* servlet (Figure 4A) was developed. This allows for a comparative analysis of multiple, related chromosome/plasmid loci of the genomic neighborhood of a single sRNA of interest (single mode) as displayed in Figure 4B. The results are translated into an image (.png-formatted) whereby homologous genes (CDS, RNA) of the sRNA locus are identified by BLASTP [12] and presented with an identical colour code. Terminators and any number of additional features previously defined can be included as desired. Each object in the image is associated with a popup-box, displaying further information and linked to corresponding database entries. The width of the resulting image can be varied to compensate for different screen resolutions. Thus one sRNA locus can be compared to different chromosomes/plasmids in a concise image output.

For the genome wide analysis of multiple sRNA loci an additional batch mode is available. Results from an application of this batch mode have already been published by Mraheil and collaborators [22]. In order to permit this global analysis an option was implemented that enables export of the data to an Excel sheet. This contains a visualization matrix (Figure 4C) which indicates the occurrence of the sRNA of interest in the target organism together with its directional relationships of the flanking genes.

The software tool presented here is a valuable extension to existing solutions and will assist in the rapid analysis of large volumes of data to understand the distribution and evolution of sRNAs in bacteria. Compared to other databases the comparative batch mode of sRNAdb’s *vision* servlet facilitates analyses such as *in silico* screening for phylogenetic markers, or identification of drug targets related to bacterial sRNAs. As exemplified by Mraheil and colleagues [22] a grouping of sRNAs from pathogenic, apathogenic or non-pathogenic bacterial strains based on the *vision* servlet´s result matrix, allows the user to identify sRNAs as putative phylogenetic markers. Specifically, sRNAs found exclusively in pathogenic strains can be identified as drug target candidates. Furthermore after download and local installation of sRNAdb, both the database and the dedicated software tools are available to the user. Since proprietary replicons or putative sRNAs can easily be included into locally installed versions of the database, these may be analysed making use of the full power of sRNAdb’s software tools, simplifying detailed analyses of unpublished bacterial replicons or sRNA candidates. To the best of the author’s knowledge, this functionality is currently not supported by any other publicly available sRNA database.

## Conclusion

sRNAdb offers biologists an easy access and analysis to both proprietary and public data and allows the identification of a core set of sRNAs which can be used as putative drug targets in antimicrobial therapeutic approaches as well as specific sRNAs for potential diagnostic markers for the detection of gram-positive bacteria.

## Availability and requirements

The database including documentation and tools for analysis are available free of charge at http://bioinfo.mikrobio.med.uni-giessen.de/sRNAdb.

## Competing interests

The authors declare that they have no competing interests.

## Authors’ contributions

Designed and implemented the database and related software tools: JP, CK, AB, TH. Analyzed data: JP, CK, AB, FC, TH. Wrote the paper: JP, CK, JH, FC, TC, TH. All authors read and approved the final manuscript.

## Funding

This work was supported by grants from the German Federal Ministry of Education and Research (BMBF ERA-NET) Pathogenomics Network to the sncRNAomics project (62080061) to T.H. and the German Centre for Infection Research, Justus-Liebig University Giessen.
